# Traffic violations analysis: Identifying risky areas and common violations

**DOI:** 10.1016/j.heliyon.2023.e19058

**Published:** 2023-08-09

**Authors:** El Mehdi Ben Laoula, Omar Elfahim, Marouane El Midaoui, Mohamed Youssfi, Omar Bouattane

**Affiliations:** a 2IACS Laboratory, ENSET, University Hassan II of Casablanca, Mohammedia, Morocco; b M2S2I Laboratory, ENSET, University Hassan II of Casablanca, Mohammedia, Morocco

**Keywords:** Traffic violations, Road safety, Kolmogorov-smirnov (KS) test, Clustering enforcements, K-means

## Abstract

Road traffic accidents caused by traffic violations are a major public health issue that results in loss of lives and economic costs. Therefore, it is important to prioritize road safety measures that reduce the incidence and severity of accidents. In this study, we suggest an incremental road safety strategy that identifies high-risk areas and common traffic violations in order to prioritize further enforcement. In fact, by analyzing data on traffic violations in different districts and comparing them to the overall average using the Kolmogorov-Smirnov (KS) test, risky areas are identified and the most common violations are detected. We performed a comparison between several types of clustering optimizations to spot clusters to be enforced in order to reduce violations. Our results indicate that some Districts have a higher risk of traffic violations than others do, and some violations (Speeding, Registration, License, Belt, Influence, Phone, etc.) are more common than others are. We also find that k-means clustering provides the best results for identifying clusters of violations records and optimizing enforcement strategies. Our findings can be adopted by law enforcement agencies to focus on high-risk areas and target the most common violations in order to optimize their resources and improve road safety.

## 1 Introduction

Road traffic accidents are a major public health concern worldwide. The World Health Organization (WHO) reports that approximately 1.35 million people die each year due to road traffic accidents, making it the leading cause of death among young people aged 5–29 years [1]. Moreover, road traffic accidents are estimated to cause economic losses of up to 3% of a country's gross domestic product (GDP) [2]. Therefore, it is essential to prioritize measures that aim to reduce the incidence and severity of road traffic accidents.

One of the key contributors to road traffic accidents is traffic rule violations such as speeding, running red lights, and reckless driving that increase the risk of accidents and fatalities on the roads [3]. To improve traffic safety, two major strategies are mainly adopted: the top-down strategy and the bottom-up approach [[4], [5], [6]]. While top-down strategies involve a comprehensive approach to risk, taking into account all possible risks and their interconnections, bottom-up ones identify a specific scope of risk (in our case, areas and types of violations) and target interventions in those areas. According to this approach, it is crucial to identify areas with high rates of traffic violations and prioritize enforcement efforts in these areas. One way to achieve this is by using statistical methods to analyze not only the statistics of violations but also their distributions over space and to compare these distributions with empirical distributions or standards [7].

The Kolmogorov-Smirnov (KS) test is a non-parametric statistical test that is widely used to compare two probability distributions. In the context of traffic safety, the KS test can be used to compare the distribution of traffic violations in a particular area with a known distribution or standard [8]. By doing so, traffic safety officials can identify areas with high rates of violations and prioritize enforcement efforts in these areas [9]. In recent years, there has been an increasing interest in the use of statistical methods, including the KS test, to improve traffic safety [10,11].

In this paper, we explore the application of the KS test to traffic safety and demonstrate its usefulness in identifying areas with high rates of traffic violations. We present a case study of using the KS test to evaluate the distribution of 13 types of violations in a geographical area and compare it with a local average to identify risky places and the most common violations in order to prioritize enforcement efforts. Finally, we compare this approach with other approaches and strategies and present its advantages in providing a comprehensive analysis of traffic safety [12].

## 2 Related works

Several studies have been conducted on using computer science technology and applied algorithms to improve traffic safety. For instance, a study by Kwon et al. (2019) used data mining techniques to analyze traffic violation data and identify high-risk areas for traffic accidents [13]. Similarly, a study by K. A. Ismail et al. (2010) proposes the use of computer vision for automated and objective traffic conflict analysis, presenting methodologies for tracking and classifying road users, measuring their real-world coordinates, detecting pedestrian-vehicle conflicts, evaluating pedestrian scramble treatments, and detecting spatial traffic violations [14]. Another study conducted by P.S. Reddy et al. suggested an automated traffic violation detection system that can accurately identify signal violations in real-time using computer vision techniques, providing efficient monitoring and enforcement of traffic regulations, surpassing the limitations of human capacity, and enabling simultaneous detection of multiple violations [15]. In addition, R.J. Franklin et al. (2020) proposed a computer vision-based system that detects traffic violations employing YOLOV3 object detection to track and penalize violations such as signal jumps, vehicle speeds, and vehicle counts. The optimized implementation achieved high accuracy: 89.24% for speed violation detection [16].

Among all these studies aiming to reduce the traffic violations and reduce traffic accident, some works consider the use of statistical methods. In fact, in their study, T. B. Ambro and others (2021) [17] aimed to identify and evaluate major traffic violations and related risk factors using multinomial logit model. The results, based on data collected in China, revealed six major traffic violations: traffic light violation, illegal parking, wrong-way driving, speeding, and not wearing a seat belt. The findings suggest that considering these risky contributing factors during traffic regulations and enforcement development can help reduce traffic violations, create a smooth/healthy driving condition, and improve traffic safety. The [18] study performed by G. Zhang and others in 2013 analyzed traffic accident data from Guangdong Province for the period 2006–2010, with a focus on traffic violations and accident severity. The study found that reducing traffic violations, targeting different vehicle types and driver groups, and improving road and transport facilities are crucial measures needed to promote road safety in China and other regions. Then, M. H. Hosseinlou and others aimed in their research [19] to identify factors that contribute to the high rate of traffic violations and crashes on Iran's freeways Using statistical models. The study analyzed data from 36 road segments and found that average speed has a positive correlation with the number of violations and crashes, while peripheral landscapes, the number of interchanges, the number of passing lanes, and exemption from paying tolls have an inverse relationship with violations and crashes.

Another field of research involves clustering traffic violations and identifying high-risk spots. In fact, S. Vardaki and al focus on Greek drivers and their self-reported tendency to commit traffic violations related to speeding, drunk driving, and cell phone use [20]. Through cluster analysis, the authors identified three distinct groups of drivers with different attitudes and behaviors toward traffic violations. The findings suggest that age, gender, and area of residence are factors that influence drivers' attitudes and behaviors toward traffic violations. Another work proposes and compares two approaches for detecting vehicular spatial violations, k-means clustering and pattern matching with the longest common subsequence (LCSS) similarity measure [21]. The results show that LCSS matching was generally superior to k-means clustering for detecting U-turn violations at an urban intersection in Kuwait City. Finally [22], explores the spatio-temporal clustering patterns of traffic collisions using network-constrained methods, which were tested on data from the Jianghan District of Wuhan, China. The proposed methods, such as weighted network kernel density estimation, network cross K-function, network differential Local Moran's I, and network local indicators of mobility association, could help researchers, practitioners, and policymakers better understand the hotspot changes and reduce the risks associated with traffic collisions.

## 3 Methodology

**Fig. 1:**
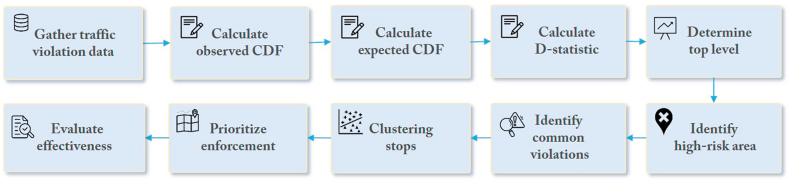
Overview of the proposed approach.

There has been an increasing concern about traffic safety and the need for effective measures to reduce traffic violations and accidents. As introduced before, a potential approach is the use of statistical methods to identify high-risk areas and prioritize enforcement efforts. One such method is the KS test [23], which can compare the distribution of traffic violations in different areas and identify those with a higher rate of violations [[24], [25], [26]]. The KS test can detect areas with higher traffic violations than the national average, making it possible to use safety systems in these locations for effective targeting of repeat offenders, reducing deployment costs and false positives. Integrating safety technologies into the methodology can overcome traditional data collection limitations and provide comprehensive traffic safety data, such as human observation and manual data entry. It can also provide a more comprehensive analysis of traffic safety by providing data on the types of vehicles involved in violations and the times and locations of the violations. Fig. 1 presents the methodology for using the KS test to detect the most problematic areas and most common traffic violations.

A Gather traffic violation data observed in different areas:

The first step is to collect traffic violation data for different areas in order to conduct an analysis of traffic safety. The data collected should include the location of the violation, the type of violation, and the identification of the offending vehicle. This information can be gathered from traffic cameras, police reports, or other sources.

B Calculate the observed cumulative distribution function (CDF):

The cumulative distribution function (CDF) is a mathematical function that describes the probability distribution of a random variable by giving the probability that the variable takes a value less than or equal to a certain point. In other words, as shown in equation (1), it tells us the probability of observing a value less than or equal to a given value. In fact, for a continuous random variable X, as is our case, the CDF is defined as:

(1) F(x)=P(X≤x)

where F(x) represents the probability that X takes a value less than or equal to x, i.e., the cumulative probability of X up to x. P represents the probability function, which gives the probability of a specific outcome or set of outcomes of X. And X represents the random variable whose CDF we are interested in. In this case, the observed cumulative distribution function (CDF) is calculated for each area based on the recorded violations and for each type of violation to detect the most problematic. This involves calculating the proportion of violations that occur at or below certain coordinates and then summing these proportions to obtain the cumulative distribution function.

C Calculate the expected CDF based on the national average distribution:

The expected CDF is calculated based on the national average distribution. This involves obtaining data on the distribution of traffic violations across the entire country and using this to estimate the expected distribution of violations in a given area. This step is essential for further comparison.

D Calculate the maximum difference between the observed and expected CDFs (D-statistic):

The D-statistic, also known as the Kolmogorov-Smirnov (KS) statistic, is a measure used in the KS test to compare two cumulative distribution functions (CDFs). The KS test is a non-parametric statistical test that determines if two datasets come from the same distribution or if they differ significantly. The D-statistic measures the maximum vertical distance between the two CDFs being compared, which represents the degree of difference between them.

**Fig. 2:**
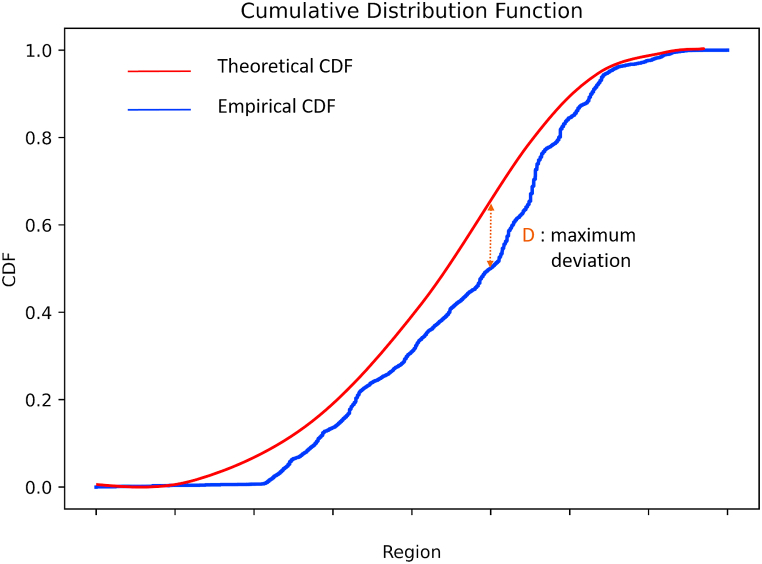
Theoretical and empirical CDF and D-statistic.

In this experiment, the D-statistic is calculated as the maximum difference between the observed and expected CDFs. As shown in Fig. 2, this parameter provides a measure of the degree to which the observed distribution of violations deviates from the expected distribution. The larger the D-statistic, the greater the difference between the two distributions. The KS test also calculates a p-value, which represents the probability of obtaining a large D-statistic or a larger one than the observed value, assuming the two datasets come from the same distribution. If the p-value is less than a predetermined level of significance (e.g., 0.05), then the null hypothesis that the two datasets come from the same distribution is rejected.

The D-statistic, presented in equation (2), is a measure of the maximum absolute difference between the cumulative distribution functions (CDFs) of two datasets. Mathematically, the D-statistic is defined as:

(2) $$D=\max\left|F_{1}\left(x\right)-F_{2}\left(x\right)\right|$$

Where F_1_(x) and F_2_(x) are the CDFs of the two datasets being compared, and max denotes the maximum value over all x values.

E Determine a significance level for the D-statistic and identify the area with the highest D-statistic:

A significance level is determined for the D-statistic to determine whether the observed deviation from the expected distribution is statistically significant. This involves comparing the D-statistic to a critical value based on the sample size and the desired level of significance. If the D-statistic is statistically significant, the area with the highest D-statistic value is identified as the high-risk area. This area is considered to have a significantly higher rate of traffic violations compared to what would be expected based on national averages.

F Identify common violation

To identify common violations, traffic violations dataset is analyzed and most common violations are identified. In fact, the spatial distribution of some stops (Speed, license, registration, traffic light, and other categories) are much more different than the average. By identifying these common violations, law enforcement agencies can focus their resources by providing enforcement: personnel designing patrols in specific areas, or equipment such as speed radars, cameras, etc.

G Clustering dataset stops

Clustering the traffic specific violations means applying clustering algorithms to identify the center of high-risk areas and optimize enforcement strategies to reduce it. We installed a cluster to be enforced for violations such as speeding and driving under the influence using tools like radar for speed and etylotest for alcohol levels. This step, by identifying the center of the problematic violations among all others, helps law enforcement agencies to allocate their resources more effectively by focusing on high-risk areas and targeting the most common violations.

In this stage, we suggest the use of following clustering optimizations.

1. **K-means** is a popular clustering algorithm that partitions a dataset into k clusters based on the Euclidean distance between the data points and the cluster centers [27]. This algorithm involves randomly initializing k centroids, assigning each data point to its nearest centroid, and then updating the centroids based on the mean of the data points in each cluster. K-means is fast, scalable, and easy to implement, but it can be sensitive to the initial centroid locations and may converge to a local minimum instead of the global minimum [28].
2. **AHL** (Agglomerative Hierarchical Clustering) is a type of clustering algorithm that is commonly used in machine learning and data mining. It is a bottom-up approach to clustering, where each data point is initially considered as a separate cluster, and clusters are then successively merged together based on their similarity [29]. In AHL, the similarity between two clusters is measured using a distance metric, and the algorithm iteratively merges the two most similar clusters until a stopping criterion is met. AHL has the advantage of being relatively simple to implement and interpret, and it can handle datasets of varying sizes and dimensions. However, it can be computationally expensive for large datasets, and the choice of distance metric can greatly affect the results of the clustering [30].
3. **DBSCAN** (Density-Based Spatial Clustering of Applications with Noise) is a clustering algorithm that groups together data points that are closely packed together and separates outliers that are far from any cluster. It defines clusters as dense regions of data points that are separated by areas of lower density. DBSCAN define a neighborhood around each data point and then grouping together points that belong to the same cluster [31]. It is robust to noise and can find clusters of arbitrary shape. However, it requires setting two hyperparameters: the radius of the neighborhood and the minimum number of points required to form a dense region [32].
H Prioritize enforcement efforts in the high-risk area:

Enforcement efforts are prioritized in the high-risk area to reduce the rate of traffic violations. License plate recognition technology can be used to identify repeat offenders and target enforcement efforts more effectively. To evaluate the effectiveness of the system in terms of reducing violations and improving traffic safety, equation (3) shows the propose Key Performance Indicator:

(3) $$KPI=\frac{NV-RV}{C}\left(TF\left(1-CF\right)\right)$$

Where *NV* is the number of violations detected before deploying the system, *RV* is the number of violations detected after deploying the system, *C* is the cost of deploying the system, *TF* is the improvement in traffic flow and reduction in congestion due to the system (measured as a percentage increase in average speed or reduction in travel time), and *CF* is a measure of the system's feasibility, taking into account factors such as public acceptance, privacy concerns, and technical challenges. The KPI formula calculates the ratio of the reduction in violations to the cost of deployment, multiplied by the improvement in traffic flow and congestion, and adjusted by the feasibility factor. A higher KPI score indicates a more effective and efficient system.

## 4 Results and discussion

### 4.1 Dataset

To understand the patterns of traffic violations in the state of Maryland, we collected a dataset that includes information on the type of violation, the location where it occurred, and the time and date of the violation for the year 2022. The data are organized by district, which allowed us to analyze the distribution of traffic violations across different areas in the state. We observed significant variations in the number of violations across the six districts of Maryland, with some districts having a much higher number of violations than others. To provide a more detailed picture of the types of traffic violations in each district, we identified different categories of violations and tagged each record accordingly.

**Fig. 3:**
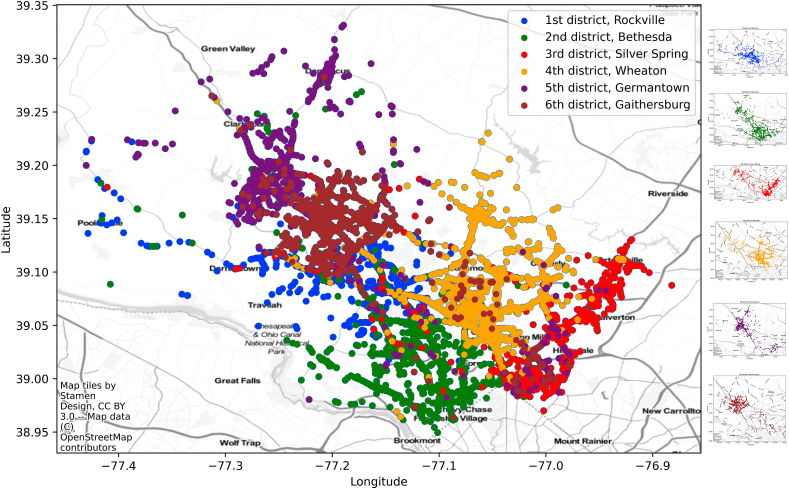
Traffic violations dataset overview.

When analyzing this data, we aim to identify high-risk areas for traffic violations and optimize enforcement strategies to improve road safety. Fig. 3 shows the distribution of traffic violations across the six districts of Maryland, USA. Each Police District violations are given with different color no matter the type of the stop.

The fields of each record are: Date Of Stop, Time Of Stop, Agency, SubAgency, Description, Location, Latitude, Longitude, Accident, Belts, Personal Injury, Property Damage, Fatal, Commercial License, HAZMAT, Commercial Vehicle, Alcohol, Work Zone, State, Vehicle Type, Charge, Article, Contributed To Accident, Race, Gender, Driver City, Driver State, DL State, and Arrest Type. To provide a more detailed picture of the types of traffic violations in each district, we identified different categories of violations and tagged each record accordingly. Table 1 shows the definition of each tag used in the dataset. These tags include failure to obey red light, exceeding speed limit, failure to register vehicle or expired registration, driving without a valid license or with a suspended license, failure to stop at a stop sign, and so on.

**Table 1:** Traffic violations type.

Traffic violation	Tag
Failure to obey red light	**Light**
Exceeding speed limit	**Speeding**
Failure to register vehicle or expired registration	**Registration**
Driving without a valid license or with a suspended license	**License**
Failure to obey device instructions, such as traffic cones or barriers	**DeviceInstruction**
Failure to stop at a stop sign	**Stop**
Using a cell phone while driving	**Phone**
Excessive window tinting or using not legal tinted materials	**Tint**
Driving under the influence of alcohol or drugs	**Influence**
Displaying a license plate that is not authorized or is obscured	**Plate**
Failure to maintain proper lane position or crossing over solid lines	**Line**
Not wearing a seat belt or allowing passengers to ride without wearing it	**Belt**
Other traffic violation	**Other**

By analyzing these tags, we were able to build separate sub-datasets for each type of violation, and for each dataset, we recorded the number of violations that occurred in each police district. This information will help us identify high-risk areas for each type of violation and optimize enforcement strategies accordingly. Table 2 details dataset stops according to their District and Type.

**Table 2:** Dataset size Traffic violations type.

Tag\District	1st Rockville	2nd Bethesda	3rd Silver Spring	4th Wheaton	5th Germantown	6th Gaithersburg	Total
**Light**	504	1012	960	1272	680	583	5011
**Speeding**	439	794	595	1225	580	470	4103
**Registration**	639	893	797	1374	459	507	4669
**License**	353	397	693	1065	330	347	3185
**DeviceInstruction**	217	756	480	619	259	273	2604
**Stop**	106	196	219	350	122	102	1095
**Phone**	109	315	142	164	74	117	921
**Tint**	49	138	87	269	153	128	824
**Influence**	113	89	172	266	95	56	791
**Plate**	102	127	208	124	57	106	724
**Line**	40	76	64	152	39	45	416
**Belt**	32	26	60	139	52	49	358
**Other**	480	828	842	1199	451	478	4278
**Total**	3183	5647	5319	8218	3351	3261	28,979

### 4.2 KS-test

**Fig. 4:**
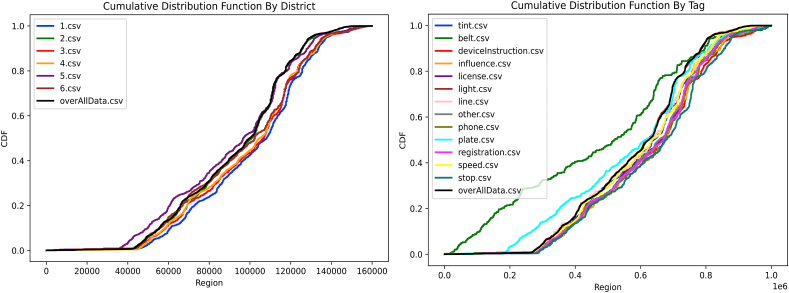
Cdf by District (a) CDF by Tag (b).

After calculating the CDF of each District a part, results, presented in Table 3, show that 1st District Rockville is the most problematic (Fig. 4-a) and that “Not wearing a seat belt or allowing passengers to ride without wearing a seat belt” is the most common violation observed followed by “Failure to stop at a stop sign” and then “influence" (Fig. 4-b).

**Table 3:** D-Statistic of traffic violation by Tag.

TAG	D-Statistic
belt	0.28768
stop	0.27445
influence	0.22853
deviceInstruction	0.22109
license	0.21334
phone	0.21464
tint	0.18677
line	0.17089
speed	0.16019
other	0.15008
registration	0.12765
plate	0.12758
light	0.03698

In contrast, violations related to “light” have a very low D-statistic of 0.03698, which indicates that these violations are more evenly distributed across the 1st ^District^ Rockville and are less indicative of high-risk areas. This information may be useful for law enforcement agencies when deciding on the allocation of resources and the types of enforcement strategies that may be most effective for reducing violations related to “light”. Therefore, law enforcement agencies could focus their resources on monitoring and enforcing “belt” and “stop” violations in these high-risk areas to help reduce the number of accidents.

### 4.3 Clustering

**Fig. 5:**
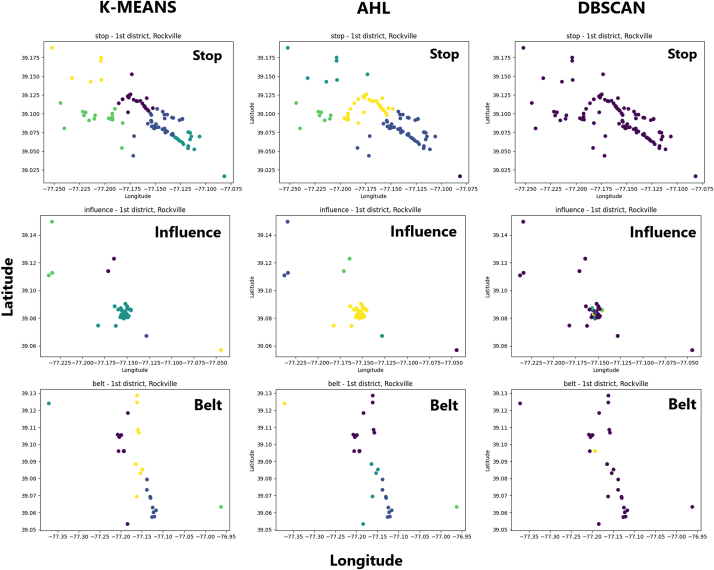
Clustering methods on common traffic violations recorded in the 1st District.

In order to further optimize enforcement strategies for enhancing road safety, we conducted a comprehensive analysis by applying three different clustering algorithms: K-means, Agglomerative Hierarchical Clustering (AHL), and DBSCAN, to identify clusters of traffic violations in the most problematic district, Rockville. Our analysis was specifically focused on the three most common violations that pose significant risks to road users, namely “Failure to stop at a stop sign”, “Not wearing a seat belt or allowing passengers to ride without wearing a seat belt”, and “Driving under the influence of alcohol or drugs”. The results of our analysis, presented in Fig. 5, reveal that K-means clustering provides the most accurate and meaningful clusters. This finding is of practical significance as it enables law enforcement agencies to efficiently allocate their resources and target high-risk areas for effective enforcement actions.

### 4.4 Discussion

In order to determine the most effective approach for improving road safety through statistical analysis of traffic violations, we compared several different methods. These methods include the before-and-after study (BAS) [33,34], time series analysis (TSA) [35,36], and the KS test, the approach used in our study. As presented in Table 4, each approach has its own advantages and limitations that should be carefully considered when selecting a method to analyze traffic violation data.

**Table 4:** Comparison between our approach, BAS and TSA.

Approach	Key Concept	Advantages	Limitations	
Before-and-After Studies [33,34]	Compare number of violations before and after implementation of intervention.	Can directly measure the effectiveness of an intervention.	Can be influenced by external factors, such as changes in traffic volume or weather conditions.	
Time Series Analysis [35,36]	Analyze changes in violations frequency over time using statistical models	Can account for trends and seasonality in violation data	-Can be complex to implement and interpret, -May require large amounts of data	
KS Test (ours)	Compare observed distribution of violations with national average using cumulative distribution function (CDF)	-Simple to implement; -Can identify high-risk areas; -Can identify high risk Tag;	-Limited to identifying areas with significantly different distributions; -Does not provide information on changes over time	

## 5 Conclusion

In conclusion, this study proposes an incremental road safety strategy to prioritize enforcement efforts by identifying high-risk areas and common traffic violations. The methodology involves the use of the Kolmogorov-Smirnov (KS) test to compare the distribution of traffic violations in different areas and identify those with a higher rate of violations. By analyzing data on traffic violations in different districts, we have identified the most problematic areas and common traffic violations. The results show that some districts have a higher risk of traffic violations than others do, and some violations are more common than others are. We also compared several types of clustering optimizations to spot clusters to be enforced, such as radars for speeding and etylotest for driving under influence. Our findings suggest that k-means clustering provides the best results for identifying clusters of violations records and optimizing enforcement strategies. Law enforcement agencies can use our findings to focus on high-risk areas and optimize their resources to improve road safety, thereby reducing the incidence and severity of accidents, saving lives and reducing economic costs.

## Author contribution statement

El Mehdi Ben Laoula: Performed the experiments; Analyzed and interpreted the data; Contributed reagents, materials, analysis tools or data; Wrote the paper.

Omar Elfahim: Performed the experiments; Analyzed and interpreted the data; Contributed reagents, materials, analysis tools or data.

Marouane EL Idaoui: Conceived and designed the experiments; Analyzed and interpreted the data.

Mohamed Youssfi: Omar Bouattane: Analyzed and interpreted the data.

## Data availability statement

Data associated with this study has been deposited at https://www.kaggle.com/datasets/rounak041993/traffic-violations-in-maryland-county.

## Additional information

No additional information is available for this paper.

## Declaration of competing interest

The authors declare that they have no known competing financial interests or personal relationships that could have appeared to influence the work reported in this paper.
